# Microsatellites explorer: A database of short tandem repeats across genomes

**DOI:** 10.1016/j.csbj.2024.10.041

**Published:** 2024-10-26

**Authors:** Kimonas Provatas, Nikol Chantzi, Michail Patsakis, Akshatha Nayak, Ioannis Mouratidis, Ilias Georgakopoulos-Soares

**Affiliations:** a Institute for Personalized Medicine, Department of Biochemistry and Molecular Biology, The Pennsylvania State University College of Medicine, Hershey, PA, USA; b Huck Institute of the Life Sciences, Pennsylvania State University, University Park, PA, USA

**Keywords:** Short tandem repeats, Microsatellites, Genomes, Human pangenome

## Abstract

Short tandem repeats (STRs) are widespread, repetitive elements, with a number of biological functions and are among the most rapidly mutating regions in the genome. Their distribution varies significantly between taxonomic groups in the tree of life and are highly polymorphic within the human population. Advances in sequencing technologies coupled with decreasing costs have enabled the generation of an ever-growing number of complete genomes. Additionally, the arrival of accurate long reads has facilitated the generation of Telomere-to-Telomere (T2T) assemblies of complete genomes. Nevertheless, there is no comprehensive database that encompasses the STRs found per genome across different organisms and for different human genomes across diverse ancestries. Here we introduce Microsatellites Explorer, a database of STRs found in the genomes of 117,253 organisms across all major taxonomic groups, 15 T2T genome assemblies of different organisms, and 94 human haplotypes from the human pangenome. The database currently hosts 406,758,798 STR sequences, serving as a centralized user-friendly repository to perform searches, interactive visualizations, and download existing STR data for independent analysis. Microsatellites Explorer is implemented as a web-portal for browsing, analyzing and downloading STR data. Microsatellites Explorer is publicly available at https://www.microsatellitesexplorer.com.

## 1 Introduction

Repetitive DNA sequences are prevalent across organismal genomes and contribute to genome architecture, evolution and function. The different types of repetitive sequences constitute more than half of the human genome [24] and represent the largest fraction of genomic DNA across most eukaryotic genomes [5]. Repeat elements are fast evolving, and are usually classified into two major classes, tandem repeats and dispersed repeats [35]. Short tandem repeats (STRs) are sequences consisting of multiple consecutive repetitions of an oligonucleotide repeat unit, typically up to six base pairs (bps) [13], [19], [37], and in certain definitions up to nine bps unit length [6]. STRs expand primarily due to slipped strand mispairing [45], [50]. Due to their heightened mutation rates STRs are highly polymorphic, representing a major source of genomic instability variation over relatively short evolutionary time frames [1], [11], [17], [42]. In humans, the mutation rates at individual STR loci can be up to 10,000 times higher than that of point mutations [42] and STR expansions are linked to a number of different diseases [21], [44], [51].

The continuing reduction in DNA sequencing costs have facilitated the generation of complete genomes for a rapidly growing number of organisms, across taxonomic groups. This trend is expected to continue with multiple international consortia being ongoing, which will provide complete, high quality genomes for additional organisms, with the goal of better capturing the genomic diversity present in nature [25], [27], [9]. Such developments have also included the sequencing of the complete human genome by the Telomere-to-Telomere (T2T) Consortium [33], facilitated by advances in long-read sequencing and adding almost 200 million base pairs of sequence which was previously missing. The Human Pangenome Reference Consortium currently contains 47 phased, diploid assemblies from a cohort of genetically diverse individuals, and is expected to increase the number of phased diploid assemblies to 350 by the end of 2024 [28]. The complete T2T genomes of other organisms have also included six non-human primate species [47] and other species of practical importance [12], [29], [41], [49], [8]. This wealth of diverse genomes provides an opportunity to annotate and centralize STRs across genomes and capture the STR diversity present in nature and in the human population in a central repository.

Previous databases on tandem repeats have included The Tandem Repeats Database, which contains tandem repeats for genome assemblies of 22 organisms [15], STRBase, a human STR DNA marker database for forensic purposes [38], MicroSatellite DataBase, a database of 37,680 genome assemblies of different organisms, SSRome a database of 6533 genome assemblies from various organisms [31], WebSTR, a population-wide database of short tandem repeat variation in humans [30] and RepeatsDB, a database of annotated tandem repeat protein structures [10]. MICdb is a database that includes 5043 bacterial and archaeal genomes [32] and PMDBase is a database dedicated to plant genomes which has 110 plant species [48]. However, there is no database to date that encompasses the set of short tandem repeats present across all available assemblies of different organisms [20], [39] and across the recently sequenced human haplotypes from diverse ancestries [28]. A central repository of STRs across all organisms, including recently sequenced T2T genomes, and across the complete genomes of different humans of diverse ancestries could facilitate their systematic research across biological problems.

**Fig. 1:**
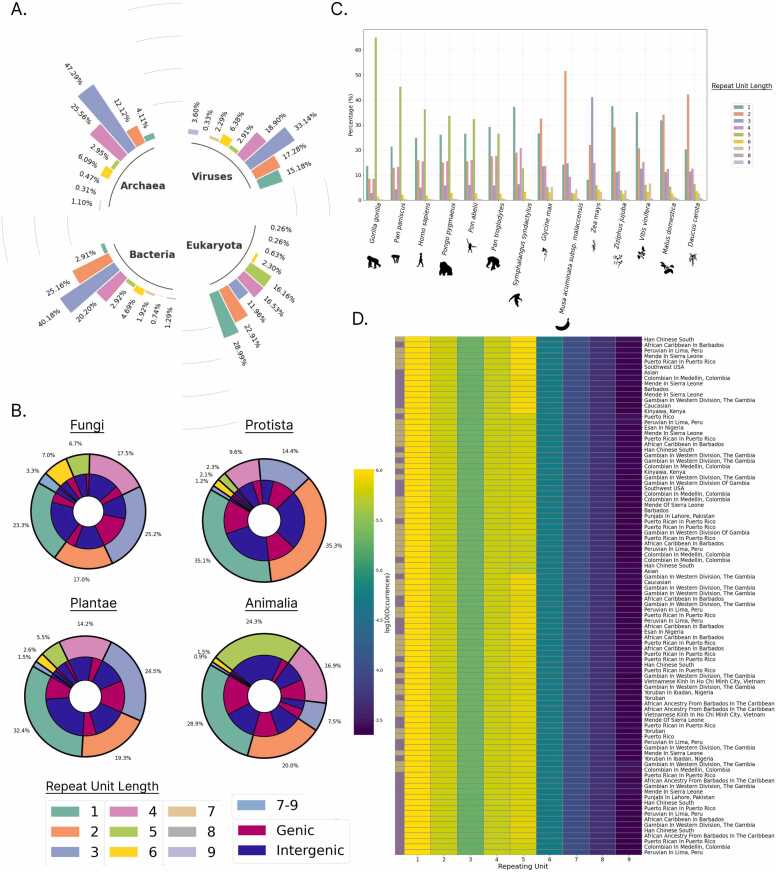
STRs found in microsatellite explorer database**. A.** Percent of STR sequences separated by domain of life and for Viruses, across the STR unit lengths. **B.** Percent of STR sequences by unit length for four eukaryotic kingdoms. **C.** Percent of STRs for each unit length across T2T genomes in the database. **D.** STR occurrences for each unit length across the Human Pangenome Project haplotypes in the database. Binary color code represents maternal and paternal haplotypes.

We recently identified STRs in 117,253 genome assemblies and 15 recently assembled T2T genome assemblies of organisms across all major taxonomic groups [7]. Here, we have expanded this dataset and incorporated 94 human T2T haplotypes of diverse ancestries and incorporated all these genome-wide STR data in a central, user-friendly database. We introduce Microsatellites Explorer, the largest database of short tandem repeats to date, spanning 117,253 complete assemblies of organisms across all major taxonomic groups (Fig. 1**A-B**), 15 T2T complete assemblies of organisms (Fig. 1**c**) and 94 human haplotypes of diverse ancestries (Fig. 1**D**). The database currently hosts 406,758,798 STRs, annotated across different organisms and human haplotypes. Microsatellites Explorer offers search options by organism ID, name and taxonomic group, STR density and multiple other characteristics. For each genome, it provides the coordinates, sequence and length of STRs identified, with options to filter and download the table. The genomes available are linked to established publicly available databases including the ENA Browser [26] and the NCBI Genome Browser [36]. We also provide an option to examine genome assemblies of multiple sub-species together. Download options enable the download of the database for further analyses.

## 2 Materials and methods

### 2.1 Data collection

STR maps were generated in [7] for all the complete genome assemblies from the GenBank and RefSeq databases [34], [4], the T2T genome assemblies from the T2T-Primates consortium [23], for the reference human genome assembly CHM13 [33] and for other T2T genome assemblies from NCBI including *Zea mays*, *Ziziphus jujuba*, *Musa acuminata malaccensis*, *Glycine max*, *Vitis vinifera, Malus domestica* and *Daucus carota*. Here, we also integrated 47 phased, diploid assemblies from a cohort of genetically diverse individuals derived from The Human Pangenome Reference Consortium [28], which were not previously described in [7], but for which you used the same methodology. STRs were detected using RPTRF, a perfect tandem repeat finder tool [3], with parameters maximum motif size, M= 50,000, and minimum length, t = 1 as described in [7].

### 2.2 Database design

The backend Flask Application is written in Python and served through reverse proxying an internal port. The data layer is handled by DuckDB in read-only mode which makes the use case of OLAP (Online Analytical Processing) fast, highly compressed, and secure. The database driver resides in-process of the Flask app memory and all data is written to a single DuckDB file (Supplementary Figure 1). The current size of the database file that supports the web application is 14 GB and holds tables with STR data and metadata (Supplementary Figure 2).

### 2.3 Web-interface

The front-end of Microsatellites Explorer is implemented in HTML, CSS, and JavaScript. The application is based on a standard Google cloud compute engine deployment that consists of a front-end app written in Javascript and served through NGINX in web server mode. A full stack web application was developed to further analyze, access, and visualize the resulting data. Dynamic and interactive graphs were created to gain a better overview of the scientific data, using custom HTML code and D3.js. Premade components were also used from the libraries Bootstrap 5 and Bootstrap 5 Datatables (Supplementary Figure 1).

## 3 Microsatellites explorer database overview and functionality

### 3.1 Database contents and usage

Microsatellites Explorer features comprehensive integration and curation of STRs in 117,253 genome assemblies of different organisms (Fig. 1**A-B**), 15 T2T genome assemblies of primate and plant species (Fig. 1**c**) and 94 human haplotypes from the Human Pangenome Project (Fig. 1**D**) in a user-friendly, interactive web interface. STR sequences can be filtered based on the STR unit length, the total STR length, and the nucleotide composition. The database includes multiple features to explore, search and download STRs from each organism or from human haplotypes in Parquet, CSV, JSON and BED formats.

**Fig. 2:**
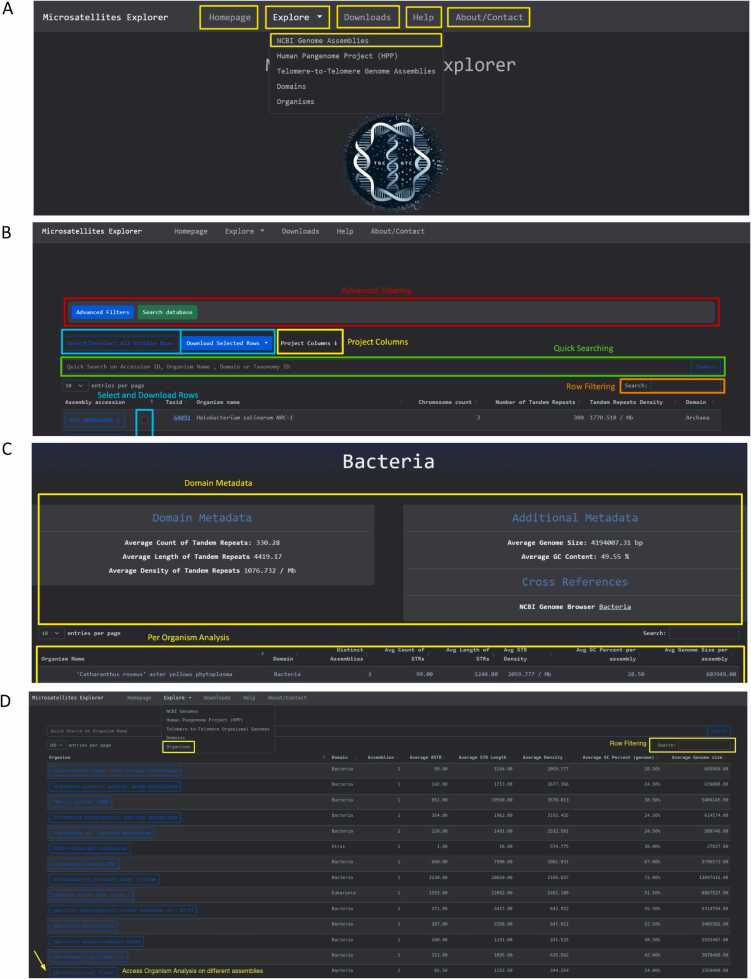
Microsatellites Explorer front-pages explaining the navigation to different pages and features**. A.** Navigation bar for dataset front-pages. The Explore page is further split into NCBI Genomes, Telomere-to-Telomere Genomes, Domains, Organisms and the Human Pangenome Project (HPP). **B.** Searching capabilities for NCBI Genomes dataset. The user can utilize the quick searching and advanced filtering options, add or remove individual columns from the table, filter rows or select rows for download. **C.** Front page of per-domain analysis. The example shown is for the domain of Bacteria. Summary information for the domain is presented. **D.** Front page for per-organism analysis. If multiple assemblies are present they are combined and STR information and statistics are provided across them.

The top navigation bar is composed of five interactive pages, namely the Homepage, the Explore page, the Downloads page, the Help page, and the About/Contact page tabs, which enable the navigation across the different parts of the database (Fig. 2**A**). The Explore page is further split into NCBI Genomes, Telomere-to-Telomere Genomes, Domains, Organisms and the Human Pangenome Project (HPP) (Fig. 2**A**). The total number of STRs across the complete genomes of NCBI organisms is 76,709,711, for T2T genome assemblies of 15 organisms is 30,491,793 and for the human HPP haplotypes is 303,016,356.

#### 3.1.1 NCBI genomes

Upon accessing the Microsatelites Explorer NCBI Genomes page, a table of the different assemblies with rich querying features enables the exploration of STRs across the genome assemblies of organisms spanning the tree of life (Fig. 2**B**). Further customization of the search is enabled by choosing specific species through the NCBI Taxonomy ID, GenBank/Reference genome accession, or species name. Advanced filtering is used to perform complex queries using all combinations of the available columns in the accession metadata (Fig. 2**B**). Additionally, the user has the option to project all the available columns from the NCBI database [40] metadata. The user can select multiple genomes for which to download STR annotations in Parquet, CSV, JSON and BED formats.

#### 3.1.2 Domains

The next aggregation of the NCBI Genome STR data is by biological domain which is provided through the navigation bar on the Explore page by selecting the Domain page. On the Domain page an analysis is performed on organisms belonging to each domain of life, namely Bacteria, Eukaryotes and Archaea as well as for Viruses. This analysis presents the average STR count, the average STR length and STR Density for each of the organism’s assemblies. Apart from the analysis per organism, STR metadata have been calculated and presented for the whole domain (Fig. 2**C**).

#### 3.1.3 Organisms

The Organisms tab in the Explore page, navigates the user to STRs grouped by species name, in which case when multiple assemblies of the species are present, there is the option to examine them together (Fig. 2**D**). Here the user can perform a quick search based on species name similarly to the NCBI Genomes search feature.

#### 3.1.4 Telomere-to-Telomere genomes

**Fig. 3:**
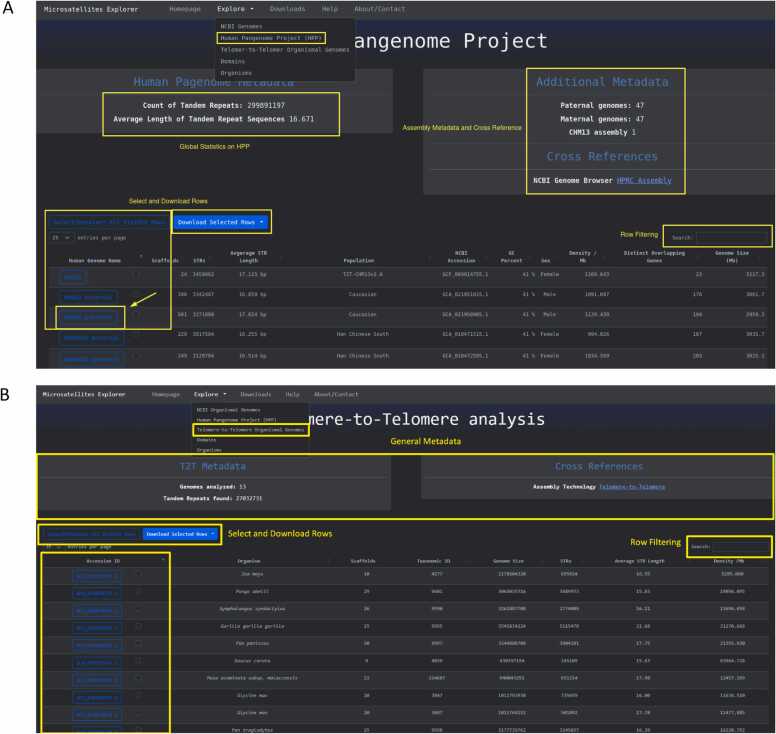
Inspect STRs in Telomere-to-Telomere genomes and in the human pangenome project**. A.** Page to inspect, browse and download Telomere-to-Telomere assemblies. **B.** Page to examine STRs across haplotypes for individuals present in the Human Pangenome Project dataset.

Advances in long read sequencing technologies have enabled the generation of high quality T2T reference genomes of different organisms. The Telomere-to-Telomere page is dedicated to these genomes (Fig. 3**A**) and provides the ability to inspect high level STR metadata along with the opportunity to select and download the data files.

#### 3.1.5 Human pangenome project

The Human Pangenome Project page consists of STR data for each haplotype and the associated metadata from the NCBI database that describe the individual human haplotype characteristics and STR summary statistics (Fig. 3**B**). Here the user can select, inspect, and download the associated human haplotype files.

### 3.2 Analysis and visualizations pages

The Microsatellites Explorer website contains interactive bar plots, pie charts, tables and drop-down menus that enable the selection and analyses of STRs across assemblies.

**Fig. 4:**
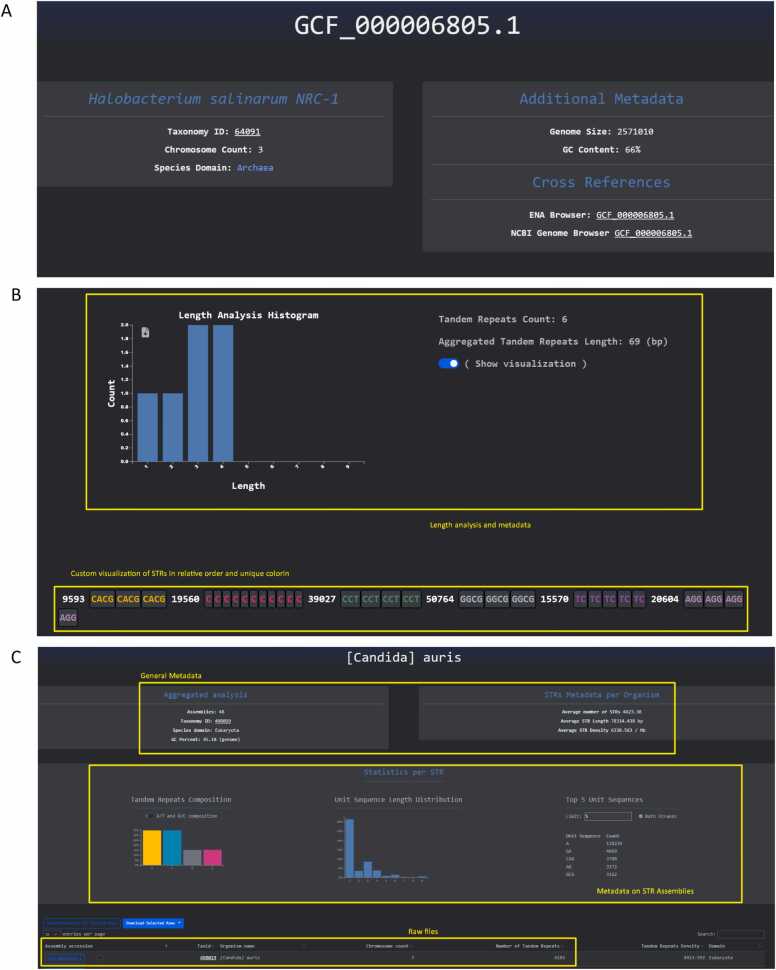
NCBI genome assembly and Human Pangenome Explore Pages**. A**. The database browser for NCBI genomes. **B.** The database browser for haplotypes from the Human Pangenome Project. **C.** When the user selects one organism they are being redirected to a web-page dedicated to the aggregation of assemblies for the organism selected. The example shown here is *Candida auris*. When viewing organismal metadata, users can explore average STR statistics and interact with dynamic graphs that illustrate STR composition, STR unit length distribution, and the top five most frequent STR sequences. Additionally, users have the option to inspect, browse, and download the assemblies associated with the organism page for further analysis.

For the Microsatelites Explorer NCBI Genomes page, when the user selects one assembly, they are navigated to a webpage dedicated to the STRs found in that genome (Fig. 4**A**). When landed on the assembly analysis page the user is presented with genomic data about the organism followed by graphs that capture relevant STR metrics. This includes bar-charts for the distribution of STRs for unit lengths of one to nine bps and the STR composition in base pairs along with the top five most frequent STR sequences. An analysis and visualization per chromosome provide the user with the ability to inspect the different STR unit lengths along with an arbitrary presentation of the unit sequences using unique coloring and a magnitude number to denote the gap between consecutive STR sequences **(**Fig. 4**B**).

**Fig. 5:**
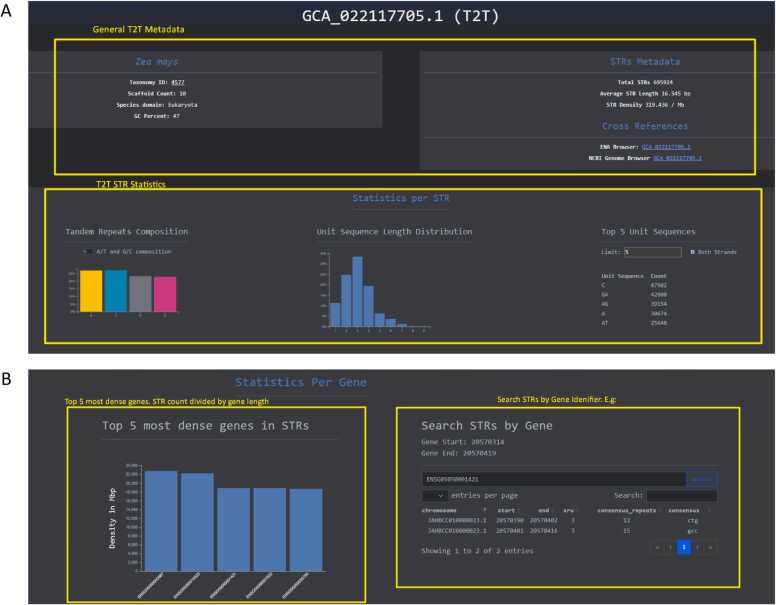
Detailed pages for the Telomere-to-Telomere genome and gene features for the pangenome assemblies**. A.** STR statistics and metadata for a selected Telomere-to-Telomere genome for *Solanum tuberosum*. **B.** Top genes ranked by STR density. Gene searching feature is shown in which the user can identify STRs by gene name or ID.

For the Organisms page, when the user selects one organism they are being redirected to a webpage dedicated to the aggregation of assemblies for the organism selected. Then the user is presented with organismal metadata, average STR statistics and dynamic graphs describing the STR composition, STR unit length distribution and the top five most frequent STR sequences. Finally, the user can inspect, browse and download the assemblies that comprise the organism page for further analyses **(**Fig. 4**C**). The same design principle is adopted for the Telomere-to-Telomere page but for each assembly, without the per-organism grouping **(**Fig. 5**A**). Regarding STR metadata and dynamic graphs the same design principle has been adopted for the Human Pangenome analysis page with the addition of search capabilities for human gene names or gene IDs along with a representation of the five most dense human genes in STRs (Fig. 5**B**).

### 3.3 About / contact, documentation, help and privacy pages

**Fig. 6:**
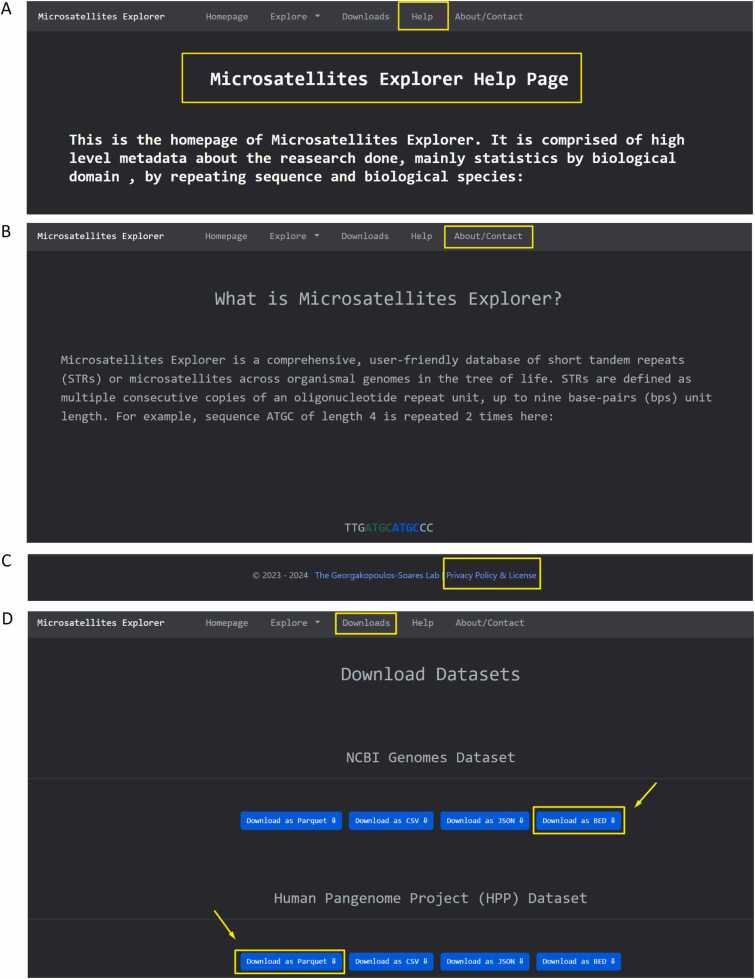
Microsatellites Explorer help, about, download and privacy& license pages**. Α.** Help Page, **B.** About Page, **C.** Privacy policy & License link to page. **D.** Download page which enables the download of each of the STR datasets in four format types Parquet, CSV, JSON and BED formats.

An About/ Contact page provides contact information for potential bug fixes and feature requests (Fig. 6A). The website has a Help page which provides information about STRs to introduce the users to the Microsatellites Explorer database and guidance to the different features of the web app (Fig. 6B). A Privacy page is integrated which includes the website’s security, policies on personal data collection and the license used which is Creative Commons 4.0 BY-SA.

### 3.4 Downloads page

All datasets can be downloaded for further processing by the user. The format of the download is a zipped folder with Parquet, CSV, JSON and BED formatted data, containing the annotated STR loci with their genomic coordinates. Data files are hosted on google cloud storage using buckets to ensure high availability and the decoupling of the app from heavy download operations (Supplementary Figure 2).

## 4 Discussion

The availability of a rapidly increasing number of genomes across different organisms and of human individuals from diverse ancestry origins provides an opportunity to collect STRs across them in a central repository, facilitating their systematic study. In this work, we present Microsatellites Explorer, a versatile and user-friendly database providing genome-wide maps of STRs across various species and the human pangenome, covering a wide range of organisms across the tree of life. Microsatellites Explorer is a comprehensive, curated database of STRs spanning 117,253 complete genome assemblies of organisms from NCBI, 15 recently sequenced T2T assemblies of different organisms and 94 haplotypes of human genomes from diverse ancestries, stored in an interactive website. As the number of available genomes continues to increase, we plan to integrate them with regular updates. The website offers multiple functionalities including interactive searches and filtering options, dynamic tables that can be queried and sorted for analysis, visualizations, data download for further analyses.

STRs have a number of functional roles including roles in gene regulation, genome organization, are associated with rapid evolutionary changes and are linked to different human phenotypes and diseases [13], [16], [18], [2], [22], [43]. Frequent mutations in STR loci can be utilized to characterize populations and identify the geographical origins of individual organisms, allowing for applications DNA forensics, PCR-based genotyping, phylogenetic analysis, ecology applications and paternity testing among others [14], [46]. Thus, the database can be utilized for genetics studies, studying genome evolution, phylogenetics, genetic diversity, molecular functions, and to use it for biotechnology applications including molecular markers.

We believe that Microsatellites Explorer will significantly improve our understanding of the roles of STRs in organismal evolution, and it has the potential to become a primary resource for studying STRs across organisms and in the human genome.

## Author contributions

K.P., N.C., and I.G.S conceived the study. N.C. and K.P. wrote the code, generated the visualizations and performed the analyses with help from M.P., A.N., I.M and I.G.S. K.P. developed the database. I.G.S. supervised the project and provided resources. K.P., and I.G.S., wrote the manuscript with help from all authors.

## Funding

Research reported in this publication was supported by the National Institute Of General Medical Sciences of the National Institutes of Health under Award Number R35GM155468. The content is solely the responsibility of the authors and does not necessarily represent the official views of the National Institutes of Health.

## CRediT authorship contribution statement

**Akshatha Nayak:** Writing – review & editing, Software, Investigation. **Nikol Chantzi:** Visualization, Software, Investigation, Formal analysis, Data curation. **Michail Patsakis:** Software, Methodology. **Kimonas Provatas:** Writing – review & editing, Writing – original draft, Visualization, Software, Methodology, Formal analysis, Data curation. **Ioannis Mouratidis:** Writing – review & editing, Project administration, Methodology. **Ilias Georgakopoulos-Soares:** Writing – review & editing, Writing – original draft, Supervision, Resources, Project administration, Methodology, Investigation, Funding acquisition, Conceptualization.

## Declaration of Competing Interest

The authors declare no conflict of interest.

## Data Availability

The Microsatellites Explorer’s dataset can be found in Zenodo with a stable version https://zenodo.org/records/13312730.
