# Supplementary material for: Microsatellites explorer: A database of short tandem repeats across genomes
Source: Comput Struct Biotechnol J. 2024 Oct 26;23:3817–26. doi: 10.1016/j.csbj.2024.10.041 (PMC11550718; doi:10.1016/j.csbj.2024.10.041)
Supplement: Supplementary file 1 — Supplementary material [file mmc1.docx]

**Supplementary Figures**

**
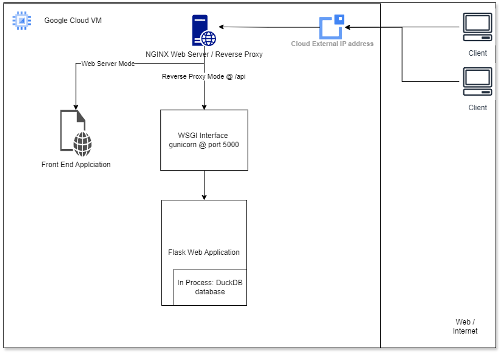
**

**Supplementary Figure 1: Cloud Deployment Diagram.** This diagram represents the deployment of Microsatellites Explorer on the google cloud platform.

**
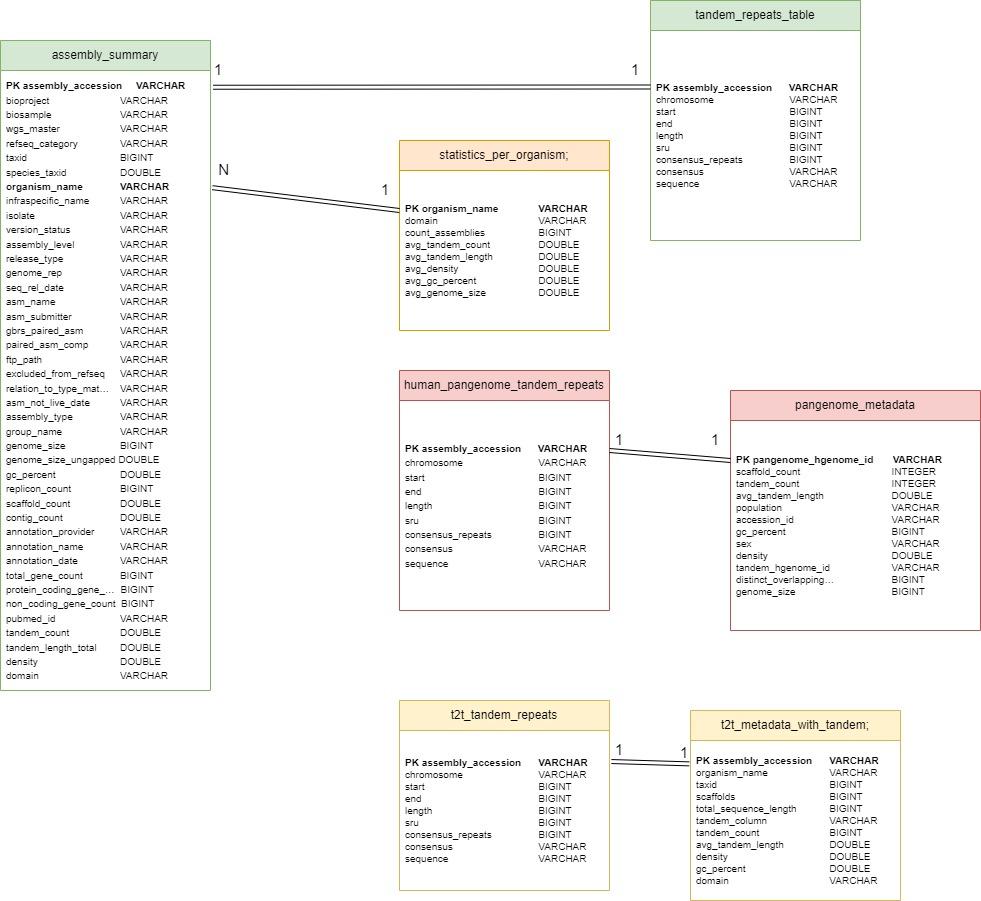
**

**Supplementary Figure 2: Database Entity Relationship Diagram.** This diagram is used to describe the column content of each database table and the cardinality relationships between entities.

**
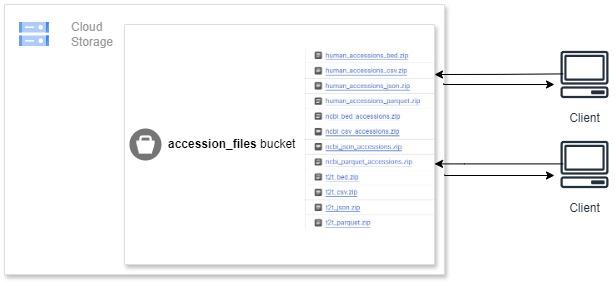
**

**Supplementary Figure 3: Storage access of dataset files.** This diagram showcases the cloud storage architecture for the hosted dataset files.
